# Influence of Vitamin D in Advanced Non-Small Cell Lung Cancer Patients Treated with Nivolumab

**DOI:** 10.3390/cancers11010125

**Published:** 2019-01-21

**Authors:** Jessica Cusato, Carlo Genova, Cristina Tomasello, Paolo Carrega, Selene Ottonello, Gabriella Pietra, Maria Cristina Mingari, Irene Cossu, Erika Rijavec, Anna Leggieri, Giovanni Di Perri, Maria Giovanna Dal Bello, Simona Coco, Simona Boccardo, Guido Ferlazzo, Francesco Grossi, Antonio D’Avolio

**Affiliations:** 1 Department of Medical Sciences, University of Turin, Amedeo di Savoia Hospital, 10149 Turin, Italy; jessica.cusato@unito.it (J.C.); giovanni.diperri@unito.it (G.D.P.); antonio.davolio@unito.it (A.D.); 2 Medical Oncology Unit, Fondazione IRCCS Ca’ Granda Ospedale Maggiore Policlinico, 20122 Milan, Italy; carlo.genova1985@gmail.com; 3 S.C. Farmacie Ospedaliere-Ospedale M.Vittoria-ASL Città di Torino, 10143 Turin, Italy; cristina.tomasello@aslcittaditorino.it (C.T.); anna.leggieri@aslcittaditorino.it (A.L.); 4 Laboratory of Immunology and Biotherapy, Department of Human Pathology, University of Messina, 98125 Messina, Italy; paolo.carrega@unime.it (P.C.); guido.ferlazzo@unime.it (G.F.); 5 Cell Factory Center, University of Messina, 98125 Messina, Italy; 6 Department of Experimental Medicine (DiMES), University of Genoa, 16132 Genoa, Italy; sele_8@hotmail.it (S.O.); gabriella.pietra68@gmail.com (G.P.); mariacristina.mingari@hsanmartino.it (M.C.M.); 7 Center of Excellence for Biomedical Research (CEBR), University of Genoa, 16132 Genoa, Italy; 8 Immunology Unit, IRCCS Ospedale Policlinico San Martino, 16132 Genoa, Italy; 9 Giannina Gaslini Institute, Via Gerolamo Gaslini, 5, 16147 Genova, Italy; ire-ne@hotmail.it; 10 Lung Cancer Unit, IRCCS Ospedale Policlinico San Martino, 16132 Genoa, Italy; erika.rijavec@hsanmartino.it (E.R.); mariagiovanna.dalbello@hsanmartino.it (M.G.D.B.); simona.coco@hsanmartino.it (S.C.); simona.boccardo@hsanmartino.it (S.B.); 11 Division of Clinical Pathology, University Hospital Policlinico G. Martino, 98125 Messina, Italy; 12 Interdepartmental Center for Clinical and Experimental Pharmacology (CIFACS), University of Turin, 10149 Turin, Italy

**Keywords:** monoclonal antibody, NSCLC, immunotherapy, ELISA, pharmacokinetics, pharmacogenetics

## Abstract

Nivolumab is one of the most commonly used monoclonal antibodies for advanced non-small cell lung cancer treatment, to the extent that the presence of its anti-antibody is considered a negative prognostic factor. Vitamin D (VD) modulates expression of the genes involved in drug metabolism and elimination. Immune system regulation and immunodeficiency is frequent in non-small cell lung cancer patients. To date, no data have been reported about the relationship between nivolumab and VD. The aim of this study was to quantify plasma 25-hydroxyVD (25-VD) and 1,25-VD, nivolumab, and its anti-antibody before starting treatment (baseline) and at 15, 45 and 60 days of therapy. VD-pathway-associated gene single nucleotide polymorphisms (SNPs) were also evaluated. Molecules were quantified through enzyme-linked immunosorbent assay, and SNPs through real-time PCR. Forty-five patients were enrolled. Median nivolumab concentrations were 12.5 μg/mL, 22.3 μg/mL and 27.1 μg/mL at 15, 45 and 60 days respectively. No anti-nivolumab antibodies were found. Correlations were observed between nivolumab concentrations and 25-VD levels. Nivolumab concentrations were affected by VD-pathway-related gene SNPs. *VDBP* AC/CC genotype and baseline 25-VD < 10 ng/mL predicted a nivolumab concentration cut-off value of <18.7 μg/mL at 15 days, which was associated with tumor progression. This is the first study showing VD marker predictors of nivolumab concentrations in a real-life context of non-small cell lung cancer treatment.

## 1. Introduction

Immunotherapy represents the most revolutionary treatment for solid cancers nowadays. To date, several types of immunotherapy are available, including monoclonal antibodies, non-specific immunotherapies, oncolytic virus therapy, T-cell therapy and cancer vaccines. The evolution of immune checkpoint inhibitors as anticancer treatment options represents one of the most successful approaches in cancer drug research in the past few years [1]. Checkpoint inhibitor antibodies, such as anti-programmed cell death protein 1 (PD-1) and its ligand (PD-L1), are new drugs acting as tumor suppressing factors since they are able to modulate the interaction between the immune cell and the tumor cell [2]. These therapies proved to be a safe and effective option in advanced non-small cell lung cancer (NSCLC) and can be recommended selectively [3]. 

Nivolumab, a monoclonal antibody, binds to the immunomodulating PD-1, blocking ligand interaction and downstream signaling pathways. The result is a positive regulation of T-cell function resulting in an antitumor effect [4]. In 2015, this drug was approved by the FDA for the treatment of patients with advanced squamous and non-squamous NSCLC with progression, or after platinum-based chemotherapy (second-line therapy) [5]. In a randomized trial, 272 patients treated with nivolumab had an overall survival of 3.2 months longer than those on docetaxel [2].

In a conference abstract, authors measured nivolumab plasma concentrations in patients and suggested that partial responders had higher nivolumab mean trough concentrations (27.4 μg/mL) compared to subjects with tumor progression (18.7 μg/mL) [6].

PD-1 inhibitors typically cause fewer and less severe treatment-related adverse events (AEs) compared with conventional chemotherapy compounds, although immunorelated AEs can occur requiring monitoring and specialized management to prevent serious complications [7]. Moreover, immunogenicity in terms of the presence of nivolumab’s anti-antibodies is considered a negative prognostic factor [8]. Immunogenicity and immune checkpoints in general are regulated by different factors such as vitamin D (VD) [9]. Reported studies show that VD controls different pathways related to innate and adaptive immunity regulating the expression of many genes involved in drug metabolism/elimination through its receptor (VDR). Moreover, in another study, single nucleotide polymorphisms (SNPs) in genes involved in the VD pathway could affect VD kinetics and, consequently, its action. Polymorphisms present near genes involved in cholesterol production, hydroxylation, and VD transport are able to predict who could have risk of VD insufficiency, as suggested by Wang et al. [10]. Genetic variations near DHCR7 (4p12 (overall *p* = 1.9 × 10(−109) for rs2282679, in GC); 11q12 (*p* = 2.1 × 10(−27) for rs12785878), near CYP2R1 (11p15 (*p* = 3.3 × 10(−20) for rs10741657) and near CYP24A1 (20q13)) have genome-wide significance in that population. Furthermore, participants with a score obtained combining the three variants in the highest quartile are at increased risk of 25-VD levels lower than 75 nmol/L or than 50 nmol/L, compared with those in the lowest quartile.

Since VD deficiency is frequent in lung cancer patients [11] and no data on nivolumab and its relationship with VD are currently available, the aim of this study was to quantify 25-hydroxyVD (25-VD), 1,25-hydroxyVD (1,25-VD), nivolumab, and its anti-antibody levels in patients’ plasma at different timings, also considering their influence in predicting the cut-off value (18.7 μg/mL) associated with tumor progression.

## 2. Results

### 2.1. Patient Characteristics

Baseline (BL) characteristics for the 45 included patients are reported in Table 1. Thirty-one (69) were male, the median age was 73 years and the median body mass index (BMI) was 23.4 Kg/m^2^.

### 2.2. Nivolumab and Vitamin D Concentrations

**Figure 1:**
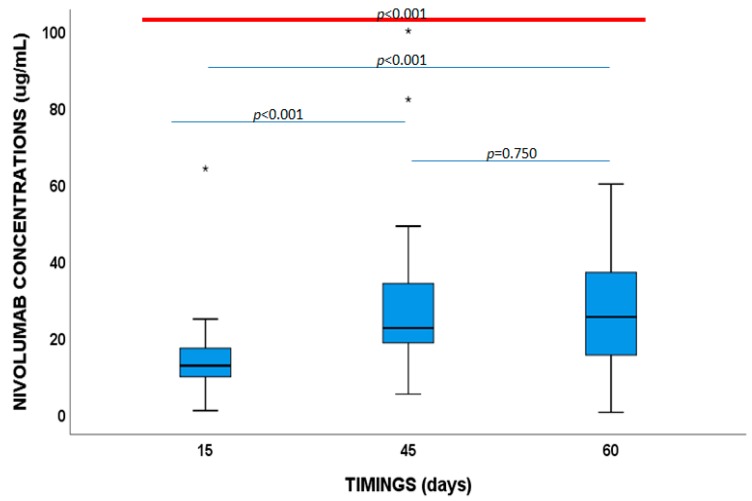
Nivolumab plasma concentrations at 15, 45 and 60 days.

Median nivolumab concentrations were 12.5 μg/mL (9.5–17.1 μg/mL), 22.3 μg/mL (IQR:18.30–34.88 μg/mL) and 27.1 μg/mL (IQR:17.4–39.4 μg/mL), respectively, at 15, 45, and 60 days (Figure 1). No anti-nivolumab antibodies were detected.

The 25-VD concentration was 12.8 ng/mL (10.1–16.6 ng/mL), 13.6 ng/mL (10.9–16.1 ng/mL), 11.8 ng/mL (10.1–18.9 ng/mL), and 12.9 ng/mL (10.8–17.0 ng/mL) at BL, 15, 45, and 60 days, respectively.

The 1,25-VD value was 33.7 pg/mL (23.4–40.6 ng/mL), 34.7 ng/mL (22.3–45.4 ng/mL), 28.5 ng/mL (20.7–41.5 ng/mL), and 35.7 ng/mL (IQR:19.2–49.0 ng/mL), respectively, at BL, 15, 45, and 60 days.

**Figure 2:**
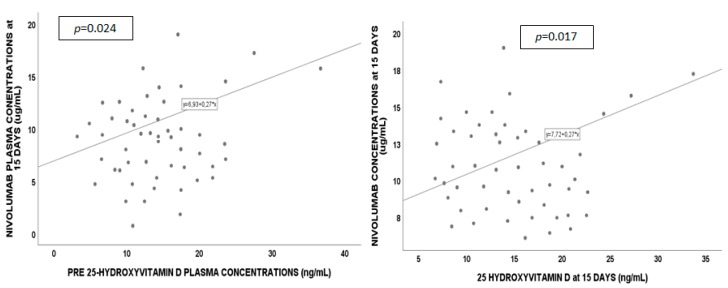

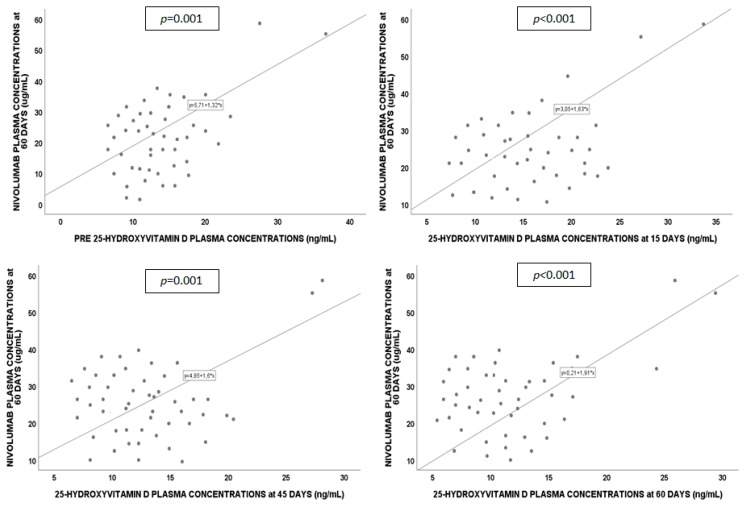
Nivolumab and 25-hydroxyvitamin D correlations at different timings.

Correlations (see Figure 2) were observed between nivolumab concentrations at 15 days and BL 25-VD levels (*p* = 0.024, Pearson’s coefficient (PC) 0.451) and at 15 days (*p* = 0.017, PC = 0.542). Nivolumab exposure at 60 days was correlated with 25-VD at BL (*p* = 0.001, PC = 0.730), at 15 (*p* < 0.001, PC = 0.858), 45 (*p* = 0.001, PC = 0.779), and 60 days (*p* < 0.001, PC = 0.900). Furthermore, in a sub-group, patients were stratified according to 25-VD deficiency. BL 25-VD levels < 10 ng/mL were associated with lower nivolumab concentrations at 15 days (*p* = 0.103, a trend without statistical significance), 45 days (*p* = 0.018), and 60 days (*p* = 0.021). Fifteen days of 25-VD < 10 ng/mL levels were associated with lower nivolumab concentrations at 15 days (*p* = 0.019), 45 days (*p* = 0.019), and 60 days (*p* = 0.028). Finally, 60 days of 25-VD < 10 ng/mL was associated with lower nivolumab levels at 60 days (*p* = 0.030). No correlation was observed for 1,25-VD or toxicities and nivolumab exposure.

### 2.3. Pharmacogenetics

Variant genotype frequencies (%) were calculated and are reported in Table 2.

### 2.4. Regression Analysis

**Figure 6:**
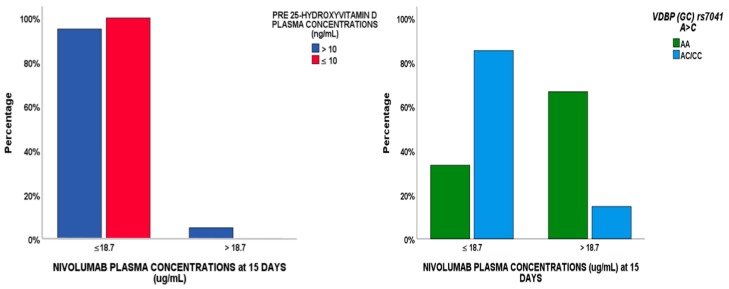
*VDBP* rs7041 SNP and pre-25 hydroxyvitamin D levels predictors of the nivolumab cut-off value of 18.7 μg/mL at 15 days, associated with tumor progression.

A logistical regression analysis was performed to evaluate whether factors (demographic, clinical, pharmacological or genetic) were able to predict nivolumab concentrations <18.7 μg/mL at 15 days (see Table 3). According to a Bonferroni test, *p* < 0.003 was considered to be the adjusted *p*-value, but no factors reached this value in the univariate analysis. In the multivariate model, *VDBP* (GC) AC/CC genotype and BL 25-VD were predictors of this cut-off value, associated with tumor progression (Figure 6).

## 3. Discussion

Nivolumab represents an active treatment strategy with the potential of long-term disease control [12]. Unfortunately, biomarkers of reliable efficacy are lacking, thus nivolumab has not been considered to be cost-effective in several national health systems [13,14].

However, a meta-analysis [3] on immune checkpoint inhibitors and chemotherapy in the treatment of advanced NSCLC showed significant advantages in terms of overall survival, progression-free survival, and overall response rate, compared with conventional chemotherapy in patients with advanced disease.

VD is able to regulate the immune system. Its synthesis begins by the action of ultraviolet light in the context of skin tissue. Cholecalciferol is hydroxylated to calcifediol (25-VD) in the liver through cytochrome P-450 (CYP, 27A1, 2R1). In the kidney, calcitriol (1,25-VD, the active form) is synthesized through CYP27B1 and transported in the bloodstream through vitamin D binding protein (VDBP). The inactivation of 25-VD to calcitroic acid (24,25-VD) is carried on by CYP24A1. VD deficiency is frequently observed in cancer patients. Bochen et al. suggested that VD serum levels were significantly lower in head and neck cancer patients compared to controls, particularly in patients with lymphatic metastasis [15]. Different studies show that a lower 25-VD serum level is associated with several negative outcomes in lung cancer. Feng et al. analyzed seventeen studies in a meta-analysis and found a statistically significant relationship between 25-VD, lung cancer risk, and mortality, but a relationship with overall lung cancer survival was not observed [16]. In addition, they suggested differences between males and females and in Caucasian and Asian populations in terms of cancer risk.

In the current study, 25-VD influenced nivolumab concentrations, but not 1,25-VD. Here, we only evaluate nivolumab and VD concentrations and not the effect on the immune cells. VD deficiency could have a relapse due to the immune system, which is directly related to this treatment. In fact, in another study, a relationship between immune cells and 25-VD and not with 1,25-VD was found, as shown for regulatory T cell function in multiple sclerosis patients [17]. Information about the influence of VD on the immune system is lacking in this study. This limitation will be the aim of further studies by our group.

Furthermore, 1,25-VD is present with a concentration 1000 times lower than 25-VD in the blood. Such low 1,25-VD concentrations could be more difficult to measure compared to 25-VD levels. Finally, the absence of statistical significance could be due to the small sample size.

In the current study, the nivolumab plasma levels in a real-life context of NSCLC are described at different timings and, in addition, the role of 25-VD concentrations and *VDBP* rs7041 A > C SNP in predicting concentrations lower than 18.7 μg/mL (the cut-off value associated with tumor progression as shown by Stijn et al. [6]) is suggested.

Various *VDBP* genetic variants are known. The two most common polymorphisms, 1296 A > C (rs7041, Glu432Asp) and 1307 C > A (rs4588, Thr436Lys), are localized in exon 11 and they are in complete linkage disequilibrium [18]. Circulating VDBP seems not to be influenced by rs7041 SNP, however, considering the 1296/1307 diplotype, there is a slight transport increase in AC/CA, compared to AA/CA. It is probable that lysine to threonine substitution at position 436 eliminates an O-glycosylation site from the molecule and the loss of glycosylation influences the half-life of VDBP. Moreover, glutamine to asparagine changes in the 432 position affect the extent of O-glycosylation at the 436. It is not known how changes in the VDBP molecule modify its serum concentration, but the described substitutions could result in altered rates of transcription, changes in mRNA stability, or in a self-clearance of the protein [19]. In a recent study of Caucasian women, the AA genotype was related to higher breast cancer risk, compared to healthy controls [20].

Controversial studies are present in the literature concerning the influence of VDBP rs7041 on VD levels. Lafi et al. show that genotypes containing the variant allele of rs7041 (TT, TG) are associated with lower 25-VD concentrations than the GG genotype, whereas Daffara et al. did not find an association in coronary heart disease patients and suggest that 25-VD levels, but not VDBP genetic status, independently predicted the presence of coronary lesions at angiography [21,22]. Also, in the current study, an association between the *VDBP* genetic variant and VD levels has been evidenced, although a borderline influence (*p* = 0.049) is present with the nivolumab cut-off value. However, the best predicting factor remains 25-VD < 10 ng/mL, as showed in the regression. It is important to understand the nature of the relationship between these variables: is the VD associated with poorer outcomes, or it could be an underlying condition? In our opinion, VD deficiency could be able to affect the outcome, since it is involved in the regulation of the immune system. Furthermore, in deficient individuals before starting therapy, the situation could be more difficult to manage and complications could be more severe (for example, concerning cachexia).

Schmid et al. showed that immunotherapy efficacy was dependent on the metastatic location [23]. For these reasons, it is very important to understand which biomarkers could predict patients with a higher probability of tumor progression.

Our study would recommend to clinicians to evaluate 25-VD levels and the *VDBP* rs7041 genotype, before starting therapy, and to quantify nivolumab concentrations at 15 days, to eventually consider a drug dosage modification or VD supplementation, reducing the risk of tumor progression. It is important to highlight that these analyses are preliminary and have several limitations: They are conducted on few individuals (only 45 patients), only one cohort is analyzed, and *VDBP* SNP has a borderline influence (*p* = 0.049).

## 4. Materials and Methods

Patients were treated with nivolumab, affected by advanced NSCLC, treated within the Italian Nivolumab Expanded Access Program (NCT02475382), and enrolled in a mono-institutional translational research study at the Lung Cancer Unit of the Ospedale San Martino (Genova, Italy). This study was approved by the Local Ethics Committee (registry number: P.R. 191REG2015). Patients were eligible if they met the following criteria: (i) cytologically or histologically confirmed advanced/metastatic NSCLC, (ii) progression after at least one line of platinum-based chemotherapy, (iii) Eastern Cooperative Oncology Group Performance Status (ECOG-PS) = 0–2, (iv) no previous treatment with immune checkpoint inhibitors, (v) any brain metastasis had to be treated and clinically stable for at least 14 days before starting nivolumab, (vi) no treatment with corticosteroids at a dose higher than 10 mg/day of prednisone or equivalent. Eligible patients received nivolumab at 3 mg/kg every 14 days, with assessment by computed tomography scan (CT-scan) every 8 weeks. Nivolumab was administered until the onset of unacceptable toxicities, patient refusal, death, or up to 96 weeks from the start of treatment. Treatment beyond tumor progression was allowed based on the investigators’ judgment, as long as clinical benefit was perceived.

Values of 25-VD and 1,25-VD were evaluated at BL and at 15, 45, and 60 days after starting therapy, with enzyme-linked immunosorbent assay technique (DRG DIAGNOSTIC, Marburg, Germany) and with LIAISON^®^ XL (DiaSorin, Saluggia, Italy), respectively. Nivolumab and its anti-antibody were quantified with validated ELISA kits (Matrix Biotek, Ankara, Turkey).

Whole blood was drawn in EDTA tubes, genomic DNA was isolated from blood samples (MagnaPure Compact, Roche, Monza, Italy), and genotypes were assessed through a real-time polymerase chain reaction allelic discrimination system (LightCycler 480, Roche, Monza, Italy). The investigated gene SNPs were: *CYP27B1* (encoding cytochrome 27B1 enzyme responsible for VD active metabolite 1,25-VD production) rs4646536 (+2838) C > T and rs10877012 (−1260) G > T, *VDR* (encoding VD receptor) rs7975232 (ApaI) C > A, rs731236 (TaqI) T > C, rs10735810 (FokI) T > C, rs11568820 (Cdx2) A > G and rs1544410 (BsmI) G > A, *CYP24A1* (encoding cytochrome 27B1 enzyme responsible for VD inactive metabolite 24,25-dyhydroxyvitamin D (24,25-VD) production) rs2248359 (3999) T > C, rs927650 (22776) C > T and rs2585428 (8620) A > G and finally *GC* (encoding VD transporter, VDBP) rs7041 A > C.

The analysis of PD-L1 was performed in 29 out of 45 patients with available tumor tissue at diagnosis using Immunohistochemistry. In particular, the PD-L1 expression was assessed manually using the rabbit monoclonal anti-human PD-L1 antibody clone 28-8 (Pharm DX DAKO, CA, USA), according to the FDA approved auto-stainer link 48 protocol. The tumor samples were defined as positive when at least 1% of tumor cells showed a strong staining according to their membrane location. All variables were tested for normality through the Shapiro–Wilk test. Normal variables were described as average and standard deviation, non-normal variables as median values and interquartile range (IQR), and categorical variables as numbers and percentages. Allele frequencies were tested for Hardy–Weinberg equilibrium. Kruskal–Wallis and Mann–Whitney tests were adopted for differences in continuous variables between genetic groups, considering statistical significance with a two-sided *p*-value < 0.05. Stepwise multivariate logistic regression analysis was performed including variables with a *p*-value below 0.2 at univariate analysis to evaluate whether factors were able to predict nivolumab levels <18.7 μg/mL at 15 days. A Bonferroni correction was performed, since an adjustment made to *p*-values is needed when several dependent or independent statistical tests are being performed simultaneously on a single data set [24].

Tests were performed using IBM SPSS Statistics 25.0 for Windows (Chicago, IL, USA).

## 5. Conclusions

In conclusion, this is the first study showing an association between VD-related biomarkers and nivolumab plasma concentrations.

In the current study, for the first time, VD deficiency seems to result in altered nivolumab clearance, as shown by different associations. It is interesting to highlight that, according to these analyses, the reduction in VD concentration was not through antibodies.

Future studies will aim to analyze the effect of VD deficiency on the immune system, for example, evaluating the immunologic profile according to VD-related biomarkers or PD-1 or PD-L1 levels and their genetic variants.

These are preliminary and limited analyses, and further studies in larger and different cohorts are needed to clarify these aspects, and to improve the knowledge in the field of the monoclonal antibody treatment used in NSCLC.

## Figures and Tables

**Figure 3:**
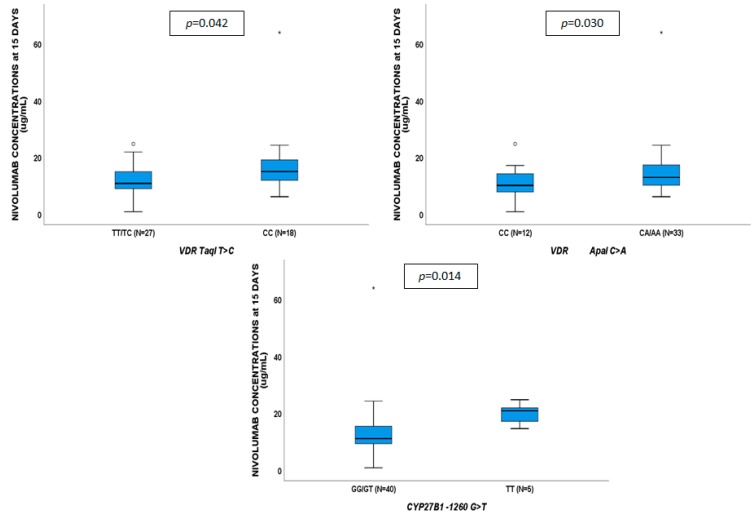
Influence of gene variants on nivolumab plasma concentrations at 15 days.

**Figure 4:**
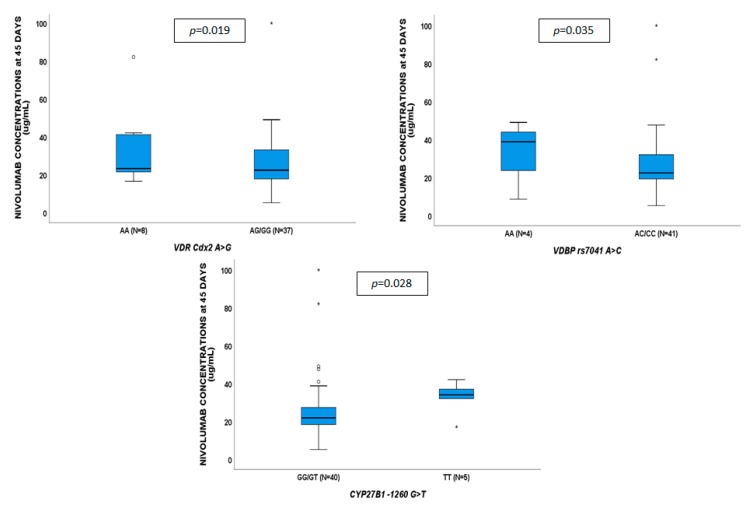
Influence of gene variants on nivolumab plasma concentrations at 45 days.

**Figure 5:**
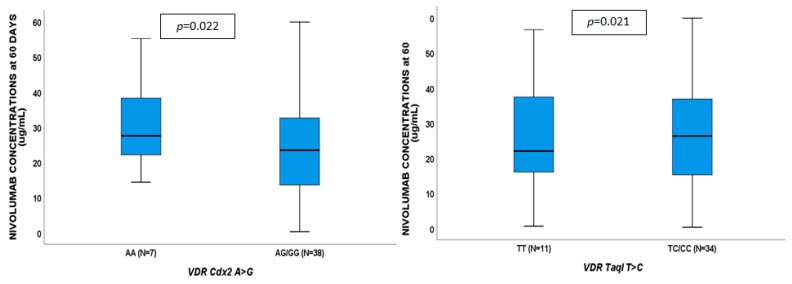
Influence of gene variants on nivolumab plasma concentrations at 60 days.

**Table 1:** Baseline characteristics of study population.

Characteristics	*n* (%), Median (IQR)	
*n*	45	
Age (years)	73 (65–79.5)	
Male sex	31 (69)	
BMI (Kg/m^2^)	23.4 (20.1–26.4)	
Caucasian	45 (100)	
NSCLC type	Adenocarcinoma	34 (52.3)
	Squamous cell carcinoma	9 (13.8)
	Poorly differentiated carcinoma	1 (1.5)
	Large-cell neuroendocrine carcinoma	1 (1.5)
Concomitant drugs	Cardiovascular	24 (36.9)
	Diabetes	4 (6.2)
	Opioids	9 (13.8)
	Protease inhibitors	20 (30.8)
	Corticosteroid	12 (18.5)
	Vitamin D	2 (3.1)
Pre-treatment drugs	Cisplatine	24 (53.3)
	Docetaxel	10 (22.2)
	Carboplatine	24 (53.3)
	Gemcitabine	12 (26.7)
	Gefitinib	2 (4.4)
	Pemetrexed	30 (66.7)
	Afatinib	1 (2.2)
	Osimertinib	1 (2.2)
	Erlotinib	20 (44.4)
	Vinorelbine	10 (22.2)
	Paclitaxel	3 (6.7)
	Bevacizumab	3 (6.7)
	Etoposide	4 (8.9)
	Zoledronic acid	1 (2.2)
	Bavicizumab	1 (2.2)
	Farletuzumab	1 (2.2)
	Radiotherapy	1 (2.2)

**Table 2:** Variant allele frequencies.

Single Nucleotide Polymorphism (SNP)	% Homozigous Wild Type	% Heterozygous	% Homozygous Mutant
*CYP27B1 +2838 C > T*	20 *CC*	2.2 *CT*	77.8 *TT*
*CYP27B1 −1260 G > T*	73.3 *CC*	15.6 *CT*	11.1 *TT*
*CYP24A1 rs2248359 T > C*	42.2 *TT*	40 *TC*	17.8 *CC*
*CYP24A1 rs927650 C > T*	33.3 *CC*	22.2 *CT*	44.5 *TT*
*CYP24A1 rs2585428 A > G*	31.1 *AA*	28.9 *AC*	40 *CC*
*VDR Cdx2 A > G*	17.8 *AA*	13.3 *AG*	68.9 *GG*
*VDR TaqI T > C*	33.3 *TT*	26.7 *TC*	40 *CC*
*VDR FokI T > C*	11.1 *TT*	42.2 *TC*	46.7 *CC*
*VDR BsmI G > A*	42.2 *GG*	57.8 *GA*	-
*VDR ApaI C > A*	26.7 *CC*	28.9 *CA*	44.4 *AA*
*VDBP rs7041 T > G*	6.7 *TT*	62.2 *TG*	31.1 *GG*

No genetic variants showed to affect VD concentrations. Nivolumab plasma concentrations at 15 days (Figure 3) were associated with *VDR* TaqI CC (*p* = 0.042), ApaI CA/AA (*p* = 0.030) and *CYP27B1*-1260 TT (*p* = 0.014). Nivolumab exposure at 45 days (Figure 4) were influenced by *VDR* Cdx2 AG/GG (*p* = 0.019), *VDBP* rs7041 AC/CC (*p* = 0.035), and *CYP27B1*-1260 TT (*p* = 0.028); nivolumab exposure at 60 days (Figure 5) was affected by *VDR* Cdx2 AG/GG (*p* = 0.022) and TaqI TC/CC (*p* = 0.021). VDR: vitamin D receptor.

**Table 3:** Logistic regression analyses: Factors able to predict nivolumab concentrations <18.7 μg/mL at 15 days of therapy. Bold represents statistically significant values. NC: not comparable, all the factors belong to a single group. Thus, statistics could show *p*-values and *odd-ratio* (OR).

Variables	Nivolumab Concentrations ≤ 18.7 μg/mL			
	Univariate		Multivariate	
	*p*-Value	OR (95% IC)	*p*-Value	OR (95% IC)
BMI < 25 Kg/m^2^	0.766	1.270 (0.392–6.112)		
Age > 60 years	0.939	0.970 (0.091–9.145)		
Gender (male)	0.213	2.260 (0.692–12.419)		
Drug dosage < 200 mg	0.945	1.056 (0.099–4.867)		
*VDBP* (GC) AC/CC	0.059	11.667 (0.909–149.700)	**0.049**	**10.667 (0.830–137.145)**
*CYP24A1* 3999 CC	NC			
*VDR TaqI TC/CC*	0.164	3.077 (0.632–14.976)		
*CYP27B1 -1260 GG*	0.148	3.250 (0.658–16.040)		
*Pre 25-hydroxyvitamin D levels*	NC		**NC**	
*Pre 1,25-hydroxyvitamin D levels*	0.124	3.840 (0.692–21.312)		
*Adenocarcinoma NSCLC type*	NC			
*Squamous cell carcinoma*	NC			
*Cisplatine pre-treatment*	0.093	4.442 (0.852–24.853)		
*Carboplatine pre-treatment*	0.190	0.300 (0.051–1.854)		
*Pemetrexed pre-treatment*	NC			
